# Improving Reproducibility and Candidate Selection in Transcriptomics Using Meta-analysis

**DOI:** 10.1177/1179069518756296

**Published:** 2018-02-27

**Authors:** Laurence A Brown, Stuart N Peirson

**Affiliations:** Sleep and Circadian Neuroscience Institute (SCNi), Nuffield Department of Clinical Neurosciences, Sir William Dunn School of Pathology, University of Oxford, Oxford, UK

**Keywords:** Meta-analysis, circadian, open science, transcriptomics, reproducibility

## Abstract

Transcriptomic experiments are often used in neuroscience to identify candidate genes of interest for further study. However, the lists of genes identified from comparable transcriptomic studies often show limited overlap. One approach to addressing this issue of reproducibility is to combine data from multiple studies in the form of a meta-analysis. Here, we discuss recent work in the field of circadian biology, where transcriptomic meta-analyses have been used to improve candidate gene selection. With the increasing availability of microarray and RNA-Seq data due to deposition in public databases, combined with freely available tools and code, transcriptomic meta-analysis provides an ideal example of how open data can benefit neuroscience research.

**Comment on:** Brown LA, Williams J, Taylor L, Thomson RJ, Nolan PM, Foster RG, Peirson SN. Meta-analysis of transcriptomic datasets identifies genes enriched in the mammalian circadian pacemaker. Nucleic Acids Res. 2017 Sep 29;45(17):9860-9873. doi: 10.1093/nar/gkx714. PubMed PMID: 28973476.

## Background

A well-planned experiment allows the collection of relevant data while minimising unwanted or unexplained variation. Variation will always exist in complex biological systems, as will a degree of variation introduced by technical issues associated with the method of data collection. With the use of new technologies, cost can also be a limiting factor in the scale of an experiment, which can lead to underpowered individual studies. The acknowledgement that no study will be ideal does not mean that the data contained within small studies are without value. Issues of reproducibility between studies have been raised in many fields of science, including neuroscience.^1^ As such, methods to combine the data from multiple studies - meta-analysis - provide a critical way of providing a scientific consensus. Meta-analysis is widely used in clinical medicine and forms one of the cornerstones of modern evidence-based medicine, on which clinical guidance and policy decisions are based. Building upon the pioneering work of epidemiologist Archie Cochrane, the Cochrane Collaboration is a good example of where routine meta-analysis is used to provide and update guidance for a range of potential medical interventions.^2,3^

Preclinical medical sciences and fundamental biological research have been slower to embrace meta-analysis. In some fields, the type of experimental techniques may vary too much for direct comparison. Moreover, data are typically not deposited on publication, making further analysis challenging. However, one field where data deposition is commonplace is in transcriptomic studies.^4^ Transcriptomics poses a number of challenging statistical issues – in particular, the high false-positive rates that arise due to the simultaneous assessment of changes in very large numbers of transcripts. Conversely, when stringent methods are used to account for false positives, transcripts that are really changing may be excluded (false negatives).^5^ Transcriptomic meta-analysis provides a valuable future avenue for addressing these problems.

## Vote Counting and Beyond

**Figure 1.**
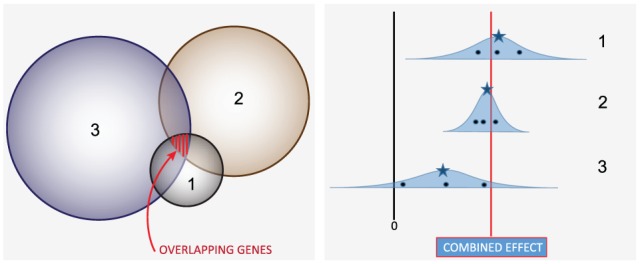
Possible approaches to meta-analysis; bringing together data from 3 different transcriptomic studies. An example of vote counting, where although the coverage of transcripts in more modern studies has improved, any agreement will be limited by those transcripts also present in earliest or lower-powered studies (left panel). Bringing together the effect sizes found in each study, placing greater confidence in those studies with lower variation (right panel).

The simplest way to look for consistent changes in gene expression across multiple transcriptomic studies is look for common transcripts among genes identified in each study. This is typically represented by the ubiquitous Venn diagram, showing overlap between studies (Figure 1). However, not all studies are equal. Some may contain larger sample sizes or less biological noise. As a result, any candidate gene list will contain false positives and will be missing false negatives that do not reach the chosen significance level for inclusion. As a result, similar to many other areas of modern life, vote counting alone may not always lead to the best-informed decisions.

By contrast with simple vote counting, meta-analysis involves applying a weighting to each study to account for factors such as sample size and biological variation. For a transcriptomic study, a simple approach to this is the use of inverse variance weighting, whereby increasing confidence is given to studies with lower variation. This provides a simple metric by which an effect size can be determined for each transcript across each study. Studies can then be compared to give a combined effect size.

## Enrichment of Transcripts in the Suprachiasmatic Nuclei

We have recently applied transcriptomic meta-analysis to identify genes involved in circadian rhythms.^6^ Circadian rhythms are ~24-hour changes in physiology and behaviour that are found in virtually all organisms. The disruption of such rhythmicity has been associated with a wide range of disorders, ranging from changes in metabolism, cardiovascular function, mental health, and even cancer.^7^ In mammals, the primary circadian pacemaker is located in the suprachiasmatic nuclei (SCN), a paired structure of 20 000 neurons in the hypothalamus.^8^ The SCN receives light input via the retinohypothalamic tract, enabling endogenous biological time to be set by the external light/dark cycle.^9^ Circadian rhythms are generated by an intracellular transcriptional-translational feedback loop comprising a number of so-called ‘clock genes’.^10^ As many of these genes show 24-hour variations in expression, a number of studies have investigated cycling transcripts in the SCN as well as peripheral tissues, to identify genes important for circadian function.^11–13^ However, the overlap between transcripts identified as rhythmic in different studies is often limited. For example, in the SCN, 2 studies identified 101 and 337 rhythmic genes, respectively.^11,13^ Of these, only 23 were common to both.^14^ A similar picture emerged in *Drosophila* microarray studies. To address this issue, Keegan et al^15^ used transcriptomic meta-analysis to reduce the false discovery rate (FDR), identifying 81 previously identified cycling transcripts, as well as 133 transcripts that were not reported in any of the previous studies.

Not all genes important for circadian physiology are expected to be rhythmic, and many may play key roles in post-transcriptional or post-translational circadian mechanisms. Furthermore, other genes may be involved in the development or intercellular communication within the SCN. Can we therefore identify genes important for circadian function via their selective enrichment in the SCN? This question led to our recent meta-analysis, based on 79 microarray and 17 RNA-Seq data sets. Our data showed enrichment of many genes known to play roles in the generation of circadian rhythms^16,17^ and previously identified markers^18,19^ but also a host of other transcripts with little or no known role in circadian biology. The limitations of such an approach must also be acknowledged. We used data from tissue from different mouse strains collected at different times across the circadian cycle. Although this may be expected to increase variance in both SCN and whole brain samples, the increased sample size still identified 426 transcripts with a combined effect size of >3 and an FDR-corrected *P* value of <.01. Such robust changes are rarely detected in individual transcriptomic studies.

## Confidence in Your Candidate: Tackling the Ignorome

The process of identifying candidate genes or transcripts that play important roles in biological processes or disease is central to modern biomedical research. In broad terms, 2 approaches have been applied to address this issue. Forward genetics, based on identifying interesting phenotypes and determining the causative gene(s), has played a critical role in many areas of research. However, over the past 2 decades, reverse genetics, moving from a gene of interest to associated phenotypes, has become much more common, primarily due to the remarkable genomic resources now available.

Transcriptomic studies provide a way of identifying potential candidate genes for subsequent study in relation to a particular biological process. However, following up a candidate gene can take months or often even years. As such, the risks of pursuing the wrong candidate have major time and financial considerations and can be especially damaging to the career of the individual researcher who is tasked with such a project. This raises a transcriptomics dilemma: how can we be sure it is worthwhile following up a specific candidate gene?

A major problem in selecting a candidate for further study is that for many genes, little is known about their function. As a result, researchers tend to focus on genes which have already been well-characterised. This is well illustrated by the power-law relationship in biomedical publication and funding, showing that researchers favour the study of relatively few genes.^20^ This is further emphasised by neuroscience studies showing that the top 5% of genes account for a remarkable 70% of the research literature. By contrast, around 20% of genes have effectively no neuroscientific literature. Such genes for which we lack any detailed biological understanding have been collectively termed the ‘ignorome’.^21^ The use of transcriptomic meta-analysis provides increasing confidence that changes in a candidate gene are real and justify further study. This in turn should encourage more research on the uncharted regions of the genome.

## The Future: Wide Open Data?

**Figure 2.**
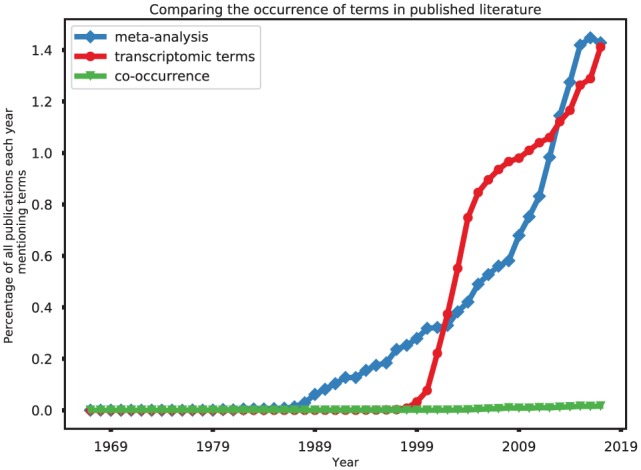
The increasing prevalence of meta-analyses and transcriptomic data in the scientific literature: even when controlling for a general increase in the volume of scientific literature, in the past 30 years, the number of scientific publications mentioning ‘meta-analysis’ each year is increasing. More recently, there has been an increasing proportion of publications that contain the terms ‘microarray’, ‘RNA-Seq’, or ‘transcriptomics’. However, transcriptomic data are the focus of less than 2% of the publications involving meta-analysis in any given year. Terms searched using https://www.ncbi.nlm.nih.gov/pubmed, on January 2, 2018 and taken as percentages of all publications in the database for said year.

The number of scientific papers published each year featuring transcriptomic data has been rising steadily (Figure 2), as well as the volume of data related to these publications (quadrillions of bases and counting).^22,23^ As a result, if a question can be addressed now using available public data, it may be possible to get a better answer next year, or even next month. Automated periodic checks for new data may also allow refinements or additional questions to be asked of the data, in the form of sub-group analysis.

It should also be the case that the methods of analysis are clear but not immutable, with incremental refinements being made over time. To aid transparency, we published our full analysis as interactive notebooks, to allow others to follow our methods. We may have even made some errors but feel it is far better to be open about this possibility than to hide our analysis from the research community. Others are taking this approach further with innovations such as the Jupyter project (https://jupyter.org/) and Binder (https://mybinder.org/)^24^ allowing for full analyses to be re-run in a Web browser. Reproducible research and open science are rightly being demanded by funding bodies and research communities alike.^25^ Luckily, the tools to facilitate these processes are improving rapidly.

Finally, it is worth stressing that transcriptomic meta-analysis is only possible because of the requirement for data deposition on acceptance of a paper for publication, along with minimal meta-data about the experimental design^4^ (although the more methodological detail the better).^23^ By contrast, widespread deposition of data in other fields does not always occur and is the first hurdle to overcome before meta-analysis becomes possible in other research areas. As well as improving candidate gene selection in transcriptomic studies, more broadly, meta-analysis provides an ideal way of improving the reproducibility and transparency of neuroscience research.
